# Acute effect of breathing exercises on muscle tension and executive function under psychological stress

**DOI:** 10.3389/fpsyg.2023.1155134

**Published:** 2023-05-25

**Authors:** Wen-Ming Liang, Jing Xiao, Fei-Fei Ren, Zi-Shuai Chen, Chun-Ri Li, Zhen-Min Bai, Osvaldas Rukšenas

**Affiliations:** ^1^Department of Neurobiology and Biophysics, Life Sciences Center, Vilnius University, Vilnius, Lithuania; ^2^Department of Physiotherapy and Rehabilitation, Xiyuan Hospital, Chinese Academy of Chinese Medical Sciences, Beijing, China; ^3^Department of Physical Education, Beijing Language and Culture University, Beijing, China; ^4^Department of Traditional Chinese Exercises, College of Physical Education, Minzu University of China, Beijing, China; ^5^Department of Acupuncture, College of Acupuncture and Moxibustion, Liaoning University of Traditional Chinese Medicine, Shenyang, China; ^6^Faculty of Health, Slovak Medical University, Banská Bystrica, Slovakia; ^7^Department of Sports Rehabilitation, School of Sports Medicine and Rehabilitation, Beijing Sport University, Beijing, China

**Keywords:** slow breathing, mindful breathing, fast breathing, music listening, muscle tension, muscle activity, executive function, cognitive function

## Abstract

**Introduction:**

Intensive and long-lasting office work is a common cause of muscular and mental disorders due to workplace stressors. Mindful and slow breathing exercises decrease psychological stress and improve mental health, whereas fast breathing increases neuronal excitability. This study aimed to explore the influence of 5 min of mindful breathing (MINDFUL), slow breathing (SLOW), fast breathing (FAST), and listening to music (MUSIC) on muscle tension and executive function during an intensive psychological task.

**Methods:**

Forty-eight participants (24 men and 24 women) were enrolled. Muscle tension was recorded using surface electromyography, and executive function was assessed using the Stroop Color and Word Test (Stroop Test). The respiration rate (RR), oxygen saturation (SpO_2_), end-tidal carbon dioxide (EtCO_2_), and the subjects' preferred method were also recorded. During the experiment, participants performed a one-time baseline test (watching a neutral video for 5 min) and then completed 5 min of MUSIC, MINDFUL, SLOW, and FAST in a random sequence. The Stroop Test was performed after each intervention, including the baseline test, and was followed by a 5 min rest before performing the next intervention.

**Results:**

None of the methods significantly influenced muscular activity and performance of the Stroop Test in both men and women, based on the average 5 min values. However, at the fifth minute, men's accuracy rate in the Stroop Test was significantly higher after SLOW than after MUSIC and FAST, and the reaction time after the SLOW was the shortest. SpO_2_ was significantly higher during SLOW than during MUSIC, and RR was relatively lower after SLOW than after MUSIC. Most men preferred SLOW, and most women preferred MUSIC, whereas FAST was the most unfavorable method for both men and women.

**Conclusion:**

Brief breathing exercises did not substantially affect muscle tension under psychological stress. SLOW demonstrated greater potential for sustaining executive function in men, possibly via its superior respiration efficiency on SpO_2_ and inhibition of RR.

## 1. Introduction

Intensive and long-lasting office work is a common cause of muscular and mental disorders due to workplace stressors (Janwantanakul et al., 2008). Many workplace stressors, such as high work and memory demands, mental load, and time pressure are risk factors for shoulder and arm pain (Bongers et al., 2002; Goh et al., 2015). One of the proposed mechanisms is that these stressors increase sustained low-level muscle activity (Bongers et al., 2006; Roman-Liu et al., 2013), which chronically increases muscle tension, impairs body alignment, causes muscular pain, and even leads to headaches (Lundberg et al., 1999; Chowdhury, 2012; Sambataro et al., 2019). In addition to increased muscle tension, chronic stress impairs cognitive function (Marin et al., 2011). Executive functions encompass cognitive processes that work for purposeful and goal-directed behavior (Banich, 2009), and perceived stress has a profound negative impact on executive functions (Kleen et al., 2006; Ohman et al., 2007). Executive function is critical for high work efficiency (Balconi et al., 2020), and high efficacy is always expected to save time. Therefore, an ideal working state should be physically relaxed and mentally effective.

It has been shown that microbreaks at work (i.e., short breaks of < 10–15 min) can benefit physical and psychological wellbeing (Henning et al., 1997; Mclean et al., 2001). Workplace physical activity significantly reduced general musculoskeletal pain, including neck and shoulder pain (Moreira-Silva et al., 2016). Three months of workplace exercise had a moderate effect on executive function compared to the control group (da Silva et al., 2021). This points to the necessity of having work breaks for physical exercise. Baduanjin exercise is one of the most common styles of traditional exercise in China. It is also a popular exercise during work breaks (Zheng et al., 2013) since it takes a short time and requires only a small space. Studies found that Baduanjin improved cognitive function and muscular performance (Zou et al., 2017; Wang et al., 2021; Yang et al., 2023). Breathing techniques are a main component of the Baduanjin exercise. In physiological studies, even a single breathing practice significantly reduced blood pressure, increased heart rate variability, oxygenated blood, and enhanced pulmonary function (Ma et al., 2017). Therefore, in order to optimize the efficacy of workplace exercises, breathing methods alone should be investigated. Slow breathing (SLOW, with a speed of 6 reps/min) and mindful breathing (MINDFUL, merely being aware of breath) are the two main breathing methods used in Baduanjin. These two breathing methods are also commonly used in yoga and psychological treatments.

Mindful breathing and SLOW can decrease psychological stress and improve mental health, while fast breathing (FAST) can increase neuronal excitability, as presented below. MINDFUL reduced emotional volatility in response to negative stimuli, reduced test anxiety (Cho et al., 2016), and even 5 min of breathing could reduce distress (Beng et al., 2016). MINDFUL increased alpha power and enhanced error-related alpha suppression during the subsequent Stroop task, indicating enhanced error monitoring (Bing-Canar et al., 2016). A study using intracranial electroencephalogram demonstrated respiration-locked oscillations during attentive breathing (mindful breathing) with stronger power in the anterior cingulate cortex, premotor cortex, insula, and hippocampus (Herrero et al., 2018), which are regions supposed to be involved in executive function (Carter et al., 1999; Rizzolatti et al., 2002; Eichenbaum, 2004). SLOW (device-guided, 5–6 breaths/min) is currently a Federal Drug Administration-approved treatment indicated by the American Heart Association for relaxation (Larson et al., 2020). Generally, for a healthy person, increased heart rate variability (HRV) indicates a relaxed state. HRV was found at the highest level when the breathing rate was 6 reps/min compared to that with spontaneous breathing and other breathing rates (Bernardi et al., 2001; Radaelli et al., 2004). Although high-frequency power in HRV (reflect vagal tone) is influenced by respiration rate (Laborde et al., 2017), many studies have found that SLOW increased ease, comfort, relaxation, and positive energy and reduced anxiety, dejection, anger, hostility, and confusion (Zaccaro et al., 2018). The executive function was improved after slow-paced breathing (4.5-s inhalation and 5.5-s exhalation) compared to after natural breathing, with higher scores observed for Stroop interference accuracy (Laborde et al., 2022). Meanwhile, the authors found that the improved executive function after slow breathing was not mediated by RMSSD in HRV using bootstrapped mediation analyses (Laborde et al., 2022). Thus, other mediating factors, such as oxygenation and ventilation volume, should be tested. In addition to MINDFUL and SLOW, FAST can increase neuronal excitability that facilitates muscle contraction and may be beneficial for cognitive functions. FAST increased ventilation and raised pH (Barrett et al., 2019). Higher pH increased Ca^2+^ and Na^+^ currents, lowered the threshold of the action potential, and shortened the refractory periods of action potentials, which facilitated muscle contraction (Tombaugh and Somjen, 1996; Lu et al., 2012). Enhanced cognitive performance was associated with increased cortical excitability, which could be related to higher glutamatergic and lower GABAergic activation (Salehinejad et al., 2021). One study compared the effect of 12 weeks of practice of slow and rapid types of pranayama breathing and found that both types of breathing were beneficial for cognitive functions, with fast pranayama having additional effects on executive function (Sharma et al., 2014).

In addition to breathing exercises, listening to music (MUSIC) is also a common way to relax during work breaks. In a survey conducted by a large North American recruitment firm, 79% of the respondents felt that MUSIC improved their work satisfaction and productivity (Haake, 2011).

To the best of our knowledge, the acute effects of different breathing techniques on muscle tension have not been investigated, and few studies have elucidated the efficacy of different breathing exercises and MUSIC on executive function. Therefore, this study aimed to explore the influence of a single 5-min session of MINDFUL, SLOW, FAST, and MUSIC on muscle tension and executive function to determine which method is better for body relaxation and mental work under psychological stress. Furthermore, the study aimed to examine physiological responses such as respiration rate, oxygen saturation level, and end-tidal carbon dioxide partial pressure.

## 2. Materials and methods

### 2.1. Participants

Forty-eight participants (men: *n* = 24, age = 30 ± 6, BMI = 23.2 ± 1.7; women: *n* = 24, age = 29 ± 6, BMI = 21 ± 1.3) were enrolled according to the estimations of sample size using G Power 3.1 (Heinrich Heine University Düsseldorf, Düsseldorf, Germany). Referring to prior studies (Springer et al., 2018; Howard et al., 2020; Redlich Bossy et al., 2020) and assuming an effect size of 0.25, alpha of 0.05, power of 0.8, and correlation coefficient of 0.6, 20 participants were required for each sex. Allowing for a 20% dropout rate, a total of 48 participants were required.

Inclusion criteria were as follows: (1) age 20–39 years; (2) BMI 18.9–24.9; (3) students or office workers; (4) ability to perform MINDFUL and SLOW (with obvious abdominal movement); (5) willingness to test at 3–10 days after the end of menstruation for women; (6) perceived stress scale ≤ 42 (Cohen et al., 1983); and (7) written informed consent. Exclusion criteria were as follows: (1) mental or cardiorespiratory diseases; (2) skeletal muscle disorders in the lower back, shoulder, or arm with a score of pain (visual analog scale) higher than 3; (3) color vision disorder; and (4) unwillingness to complete the experiment. In addition, participants were asked not to consume caffeinated drinks for 2 h before the experiment.

The study was approved by the Medical Ethics Committee of Xiyuan Hospital, China Academy of Chinese Medical Sciences (approval number: 2022XLA013-2).

### 2.2. Interventions

Mindful breathing, SLOW, FAST, and MUSIC were the four interventions. For MINDFUL, participants were required to be aware of the breath, without controlling the speed and depth, and without judgment of the quality (Burg and Michalak, 2011). The breath counting method was allowed for participants who felt it challenging to focus on their breath. SLOW was set at a speed of six breaths per minute, and obvious abdominal movement was required. An audio metronome was designed with a rising tone for inhalation and a falling tone for exhalation, setting the rate at 4 s for inhalation and 6 s for exhalation. Participants were instructed to gently expand their abdomen in addition to the natural expansion of the ribcage during inhaling, and relax their abdomen with attention on the area around the navel during exhaling. They were allowed to hold their breath naturally after exhalation to mimic natural breathing. The assessment of SLOW with obvious abdominal movement was conducted using two respiration belts. To determine the FAST rate, a respiration belt was used to measure RR during spontaneous breathing. FAST was then set at a speed that was 30% higher than an individual's regular breathing rate. During FAST, participants were also guided by an audio metronome. Regarding MUSIC, participants chose the relaxing music they preferred. Classical music (piano, Kiss the Rain; guzheng, Yun Shui Chan Xin) was provided, although participants could also listen to their own relaxing music.

### 2.3. Experimental protocol

**Figure 1:**
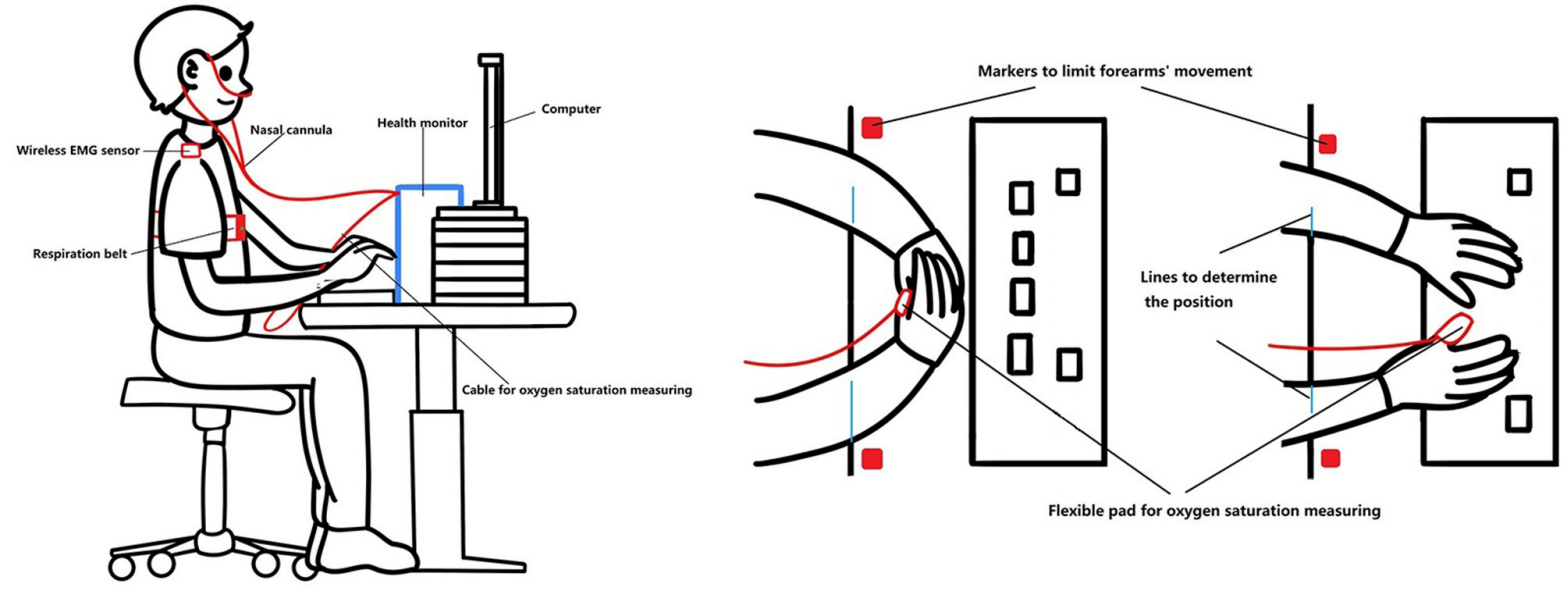
Postures during the experiment.

**Figure 2:**
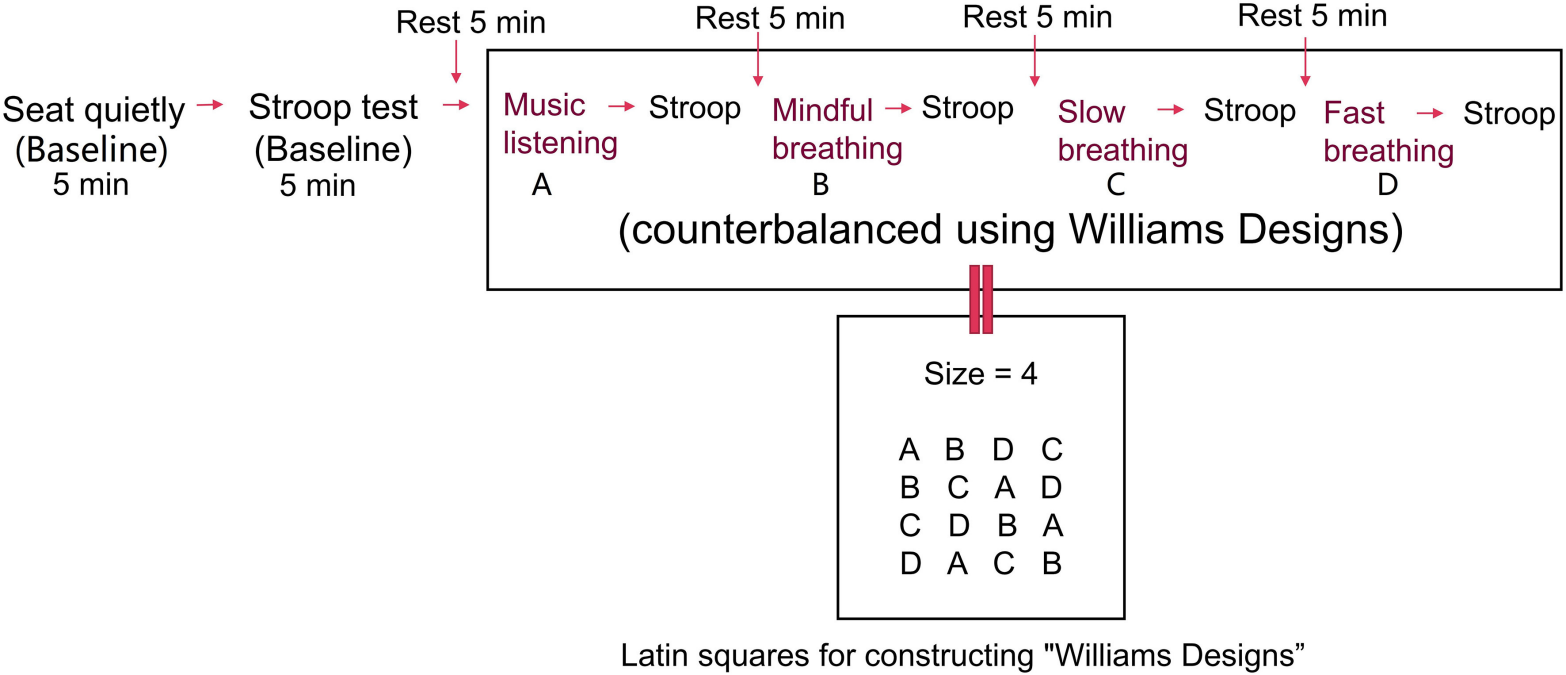
Experimental procedure.

The same participants were tested in all conditions to limit inter-individual differences and minimize random noise (Laborde et al., 2017). The duration of the interventions, the Stroop test, and the rest between interventions were set at 5 min according to the previous studies (Endo et al., 2013; Beng et al., 2016; Kluger and Gross, 2020). To ensure that the participants could perform breathing exercises properly, they received one-time online training 1 month before the testing day and were requested to practice for 1 month. All participants were invited to a social platform group where they could ask questions and exchange training videos, and instructors checked and guided participants' movement of SLOW during the 1-month training period through video recordings and/or online meetings. Prior to the experiment, participants underwent two training sessions on the Stroop test to reduce the learning effect. Then, participants sat on an adjustable chair in a standardized posture, with ~90 degrees flexion of the knees and hips, and a straight upper body (Figure 1); the computer screen was set at 50–80 cm in front of the face. The angle between the arm and the trunk was about 20 degrees, and the angle between the forearm and the arm was about 110 degrees, the junction of the front one-third of the forearm and the back two-thirds (the tester made marks on participants' arms) was above the edge of the table. Two markers were put on the table beside the participants' forearms to eliminate the movement. During the experiment, participants performed a one-time baseline test (watching a neutral video for 5 min) and then completed 5 min of MUSIC, MINDFUL, SLOW, and FAST in a random sequence. The Stroop test was performed after each intervention, including the baseline test, and was followed by a 5 min rest before performing the next intervention. While watching the video, listening to music, and performing breathing methods, participants overlapped their hands and rested the ulnar side on the table with palms toward the body and eyes closed. Before the Stroop test, participants opened their eyes and waited for 10 s to “wake up.” At the resting time, participants were allowed to stand up, walk, and drink water. Sequence randomization used the Latin Square Williams design, as shown in Figure 2.

### 2.4. Data collection

#### 2.4.1. Muscle tension

Muscle tension/activity was measured using sEMG (Delsys Trigno wireless system, Natick, MA, USA). The skin was gently abraded and cleaned with an alcohol wipe, and the hair was removed when necessary. Two rectangular Delsys Trigno EMG sensors (27 mm × 37 mm × 15 mm) with 99% silver electrode contacts adhered to the upper trapezius (halfway along the line from the acromion to the spine on vertebra C7 on both left and right sides). We chose the upper trapezius muscle because its activity is more evident than that of most other body sites during mental tasks (Schleifer et al., 2002). The wireless electrodes were suitable for our experiments since participants reported that the electrodes did not cause distraction. The sampling frequency was 2148 Hz, with an amplification gain of 1000. Raw sEMG data were band-pass filtered between 20 and 450 Hz using a fourth-order Butterworth filter, and band-stop filtered at 50 Hz with aliases with sidebands of 0.25 Hz. The RMS was used to represent muscle tension.

To verify and consolidate the results, we used two methods to normalize the RMS values. The first used the mean value (average of four interventions for each participant) as a reference. Halaki and Ginn (2012) summarized that using the mean activation level of EMG could decrease the variability between individuals. The second used the baseline (Figure 2) as the reference. Roman-Liu et al. (2013) conducted a study that tested muscle tension (using sEMG) under mental load with repeated measures and claimed that taking the baseline as the reference value was the most appropriate reference. Normalization was performed by dividing muscle tension during the baseline condition by muscle tension during different breathing methods or the Stroop test and multiplied by 100.

#### 2.4.2. Executive function

Executive function was determined from the accuracy and reaction time of the Stroop test and tested on a computer using E-prime 3.0 (Psychology Software Tools, Pittsburgh, PA, USA). The test consisted of four words (“red,” “blue,” “green,” and “yellow”), which were randomly displayed with a color that was the same as or different from the word's meaning. A series of color words were presented consecutively during the Stroop test. The participants were instructed to name the color of the displayed word as accurately and quickly as possible. A new color word was shown for 21 ms after one response or for 2000 ms without a response (no response within 2000 ms was recorded as an incorrect answer). There were 24 samples in each cycle, and the system randomly generated the sequence for every cycle. The accuracy was calculated by dividing the number of correct answers by the total number of color words and then multiplying the result by 100.

#### 2.4.3. Respiration rate

A respiration belt (Vernier, Beaverton, OR, USA) was secured around the chest, level with the xiphoid, to record the respiration rate. This belt has been used in previous studies to provide ground truth data (Jakkaew and Onoye, 2020). The signals were presented as a force with a sampling frequency of 10 Hz. There was a channel detecting inhalations and calculating the number of breaths per minute. The sample window for the calculation was 30 s, the advance interval was 10 s, and the value updates every 10 s.

#### 2.4.4. Oxygen saturation and end-tidal carbon dioxide partial pressure

A health monitor (Contec-CMS8000, Qinhuangdao, China) was used to record SpO_2_ and EtCO_2_. The SpO_2_ measuring range was 0–100%, resolution 1%, and actualization interval 1 s. The flexible pad was worn on the right thumb since the participants needed the index and middle fingers to press the keys on the keyboard. The expired air was continuously collected using a disposable nasal cannula for measuring EtCO_2_. The sampling gas flow rate was 50 ml/min ± 10 ml/min, resolution 0.1 mmHg (0–69 mmHg), actualization interval 1 s, and the delay time 2–3 s. Atmospheric pressure was set at a default value of 760 mmHg. Routine calibration was not required, but 1–2 min of warm-up was implemented prior to each participant. Participants could opt out of the EtCO_2_ test if the cannula was uncomfortable.

#### 2.4.5. Preferred intervention

At the end of the test, participants selected their preferred intervention according to their general subjective feelings of relaxation and working efficiency.

### 2.5. Outcomes

The primary outcomes were as follows: muscle tension, represented by root mean square (RMS) values from surface electromyography (sEMG) recording; and executive function, represented by accuracy and reaction time from the Stroop test. The secondary outcomes were RR from the respiration belt, SpO_2_ and EtCO_2_ from a health monitor, and the participants' preferred method.

### 2.6. Statistical analysis

The Shapiro–Wilk test was performed to assess data distribution. The percentage of missing data was calculated, and Little's MCAR test was used to determine the missingness model. The effect of interventions from the average 5 min values and every minute's values was tested using generalized estimating equations (GEEs) on SPSS (IBM Corp., Armonk, NY, USA) since normal and skewed distributions were mixed throughout the data, and some could not be converted to a normal distribution using log transform. The exchangeable correlation structure was chosen as the present study had a balanced design, and the value of goodness-of-fit was the highest. Significance was set at an alpha level of *P* < 0.05 and a highly significant level of *P* < 0.01, with the Holm correction, applied for the *post-hoc* test. The Wilcoxon paired test was used to evaluate the difference between the parameters when performing interventions and those while taking the Stroop test, and the significance was set at *P* < 0.05. Effect sizes were calculated using Hedges' g (g) method. Participants' preference (percentage of favorable intervention) was analyzed using the chi-squared test on MedCalc (MedCalc Inc., Mariakerke, Belgium), and the significance was set at *P* < 0.05 and *P* < 0.01 with Holm correction.

## 3. Results

According to the inclusion criteria, 48 participants were enrolled in this study. Three male participants were excluded: one fell asleep during SLOW and MINDFUL, one was in bad condition (the error rate was over 40% in the Stroop test), and one did not perform MINDFUL and SLOW as required. Two women were excluded: one reported a bad condition during the testing and another did not perform MINDFUL and SLOW as required. Therefore, 21 men and 22 women were included in the analysis. All missing data were missed completely at random (Little's MCAR test, *P* > 0.05).

According to the Latin Square Williams design (Figure 2), the participant performed each method according to the horizontal sequence as the first participant performed ABCD, the second performed BCAD, the third performed CDBA, the fourth DACB, and the fifth restarted from ABCD. In order to test for the effect of fatigue and/or learning, we compared each vertical sequence (ABCD, BCDA, DABC, and CDAB) using the averaged 5 min values. Every *post-hoc* comparison's result was not significant as the lowest *p*-value was 0.127 from men's data and 0.074 from women's data. Therefore, we concluded that fatigue and/or learning effects were non-significant.

### 3.1. Muscle tension

Among the included participants, the sEMG signals of one man and two women were not recorded successfully. Thus, 20 men and 20 women were included in the analysis. Men's data were skewed after SLOW and FAST, while women's were normally distributed. Men had no missing data from the right upper trapezius and 2.5% from the left side**;** women had 2.5% missing data from the right side and no missing data from the left side. Outliers were defined as outside of median – (3 × median absolute deviation) and median + (3 × median absolute deviation) (Trevino et al., 2015). Outliers were not removed because they were genuine observations. To verify the results, we analyzed the data after removing the outliers and found no significant differences from the results that retained outliers.

As depicted in Table 1, for both men and women, different interventions did not significantly affect muscle tension during the interventions and the Stroop test. However, there was a clear trend for men to have the lowest muscle tension during MINDFUL, and the tension decreased minute-by-minute. The muscle tension was relatively high after SLOW. The muscle tension in women was lower after MUSIC and FAST than that after MINDFUL and SLOW, and it was the lowest after FAST. In addition, the results were inconsistent between the left and right sides in both men and women.

**Table 1:** Results of muscle tension.

**Interventions**	**1st min**	**2nd min**	**3rd min**	**4th min**	**5th min**	**Averaged 5min**	**Wald χ^2^**	***P***
**Men's muscle activity of the right upper trapezius during the Stroop test (normalized RMS, %)**								
Music	92 ± 25	89 ± 27	95 ± 27	97 ± 28	94 ± 29	93 ± 27	3.469	0.325
Mindful	98 ± 35	95 ± 36	89 ± 33	88 ± 34	86 ± 30	91 ± 33		
Slow	95 ± 25	102 ± 29	109 ± 33	104 ± 33	104 ± 36	103 ± 31		
Fast	116 ± 58	111 ± 47	110 ± 40	110 ± 49	116 ± 56	113 ± 49		
**Men's muscle activity of the left upper trapezius during the Stroop test (normalized RMS, %)**								
Music	92 ± 34	91 ± 29	103 ± 34	107 ± 38	109 ± 38	101 ± 35	1.630	0.563
Mindful	93 ± 29	92 ± 36	96 ± 43	93 ± 42	88 ± 31	92 ± 36		
Slow	105 ± 28	109 ± 39	109 ± 36	107 ± 36	106 ± 33	107 ± 34		
Fast	110 ± 51	101 ± 37	97 ± 36	95 ± 40	94 ± 39	99 ± 41		
**Women's muscle activity of the right upper trapezius during the Stroop test (normalized RMS, %)**								
Music	98 ± 26	99 ± 26	99 ± 26	99 ± 27	99±	99 ± 26	4.249	0.488
Mindful	98 ± 39	106 ± 34	112 ± 40	116 ± 38	109 ± 35	108 ± 37		
Slow	102 ± 36	101 ± 37	104 ± 39	104 ± 37	103 ± 40	103 ± 37		
e Fast	93 ± 38	87 ± 36	90 ± 37	92 ± 37	90 ± 37	90 ± 36		
**Women's muscle activity of the left upper trapezius during the Stroop test (normalized RMS, %)**								
Music	95 ± 28	95 ± 29	94 ± 28	97 ± 27	97±	96 ± 27	1.661	0.646
Mindful	101 ± 39	103 ± 37	105 ± 34	105 ± 33	102 ± 31	103 ± 34		
Slow	113 ± 39	104 ± 36	104 ± 35	105 ± 35	106 ± 35	106 ± 35		
Fast	98 ± 34	93 ± 30	92 ± 28	95 ± 28	96 ± 30	95 ± 29		

MUSIC, listening to music; MINDFUL, mindful breathing; SLOW, slow breathing; FAST, fast breathing.

The two normalization methods yielded similar results, as both had non-significant *p*-values and similar trends.

### 3.2. Executive function

Data from 21 men and 22 women were analyzed for executive function, with no missing data. The accuracy data for men and women were skewed, while reaction times were normally distributed.

**Figure 3:**
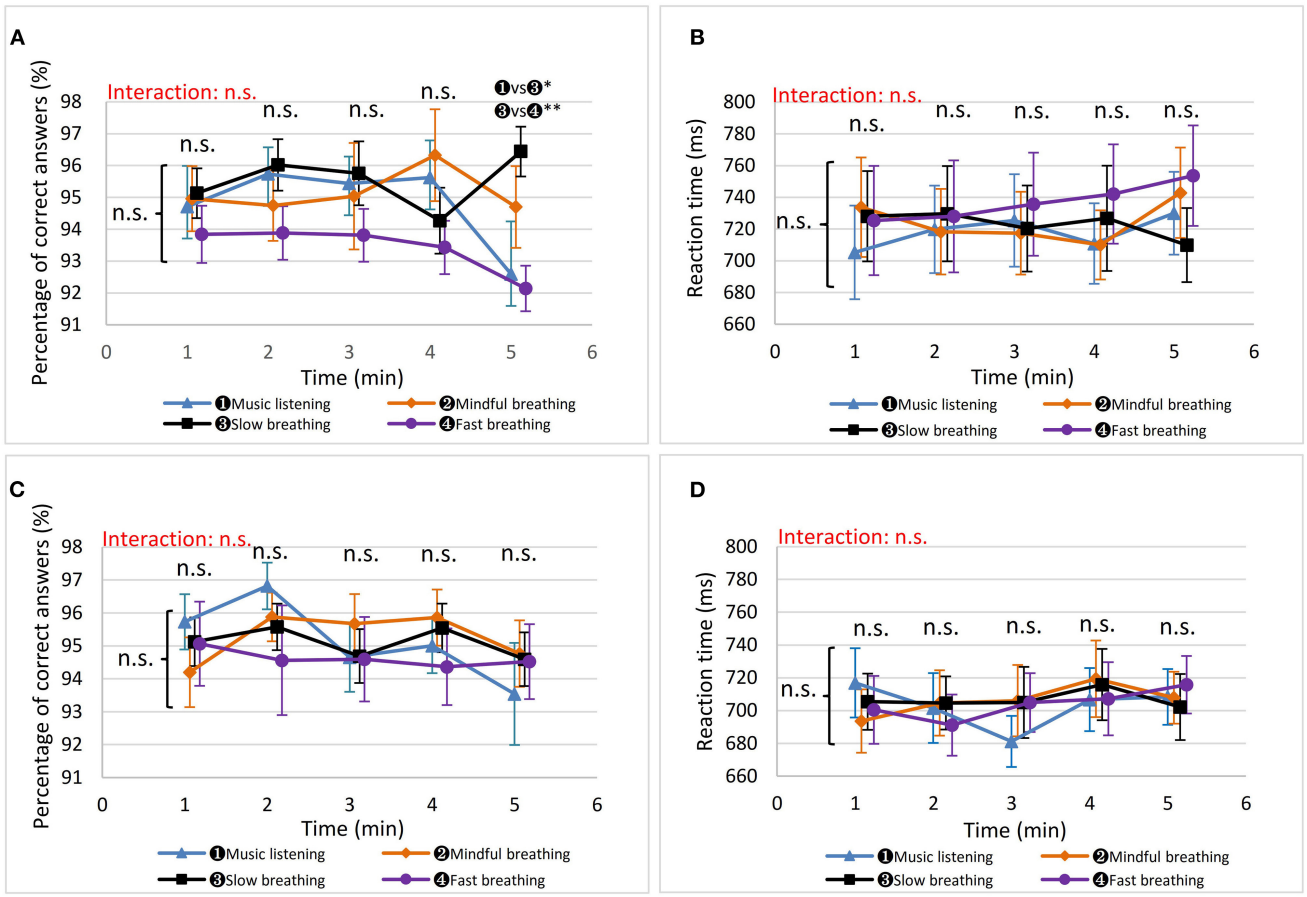
The results of executive function. “n. s.” beside the curly brackets denotes that no significant difference was found between each intervention based on the average 5 min values. “n. s.” or “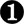 vs 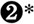
” above each minute presents the results of the comparisons based on the values in that minute. The results of interactive effects were colored in red. ^*^P < 0.05, ^**^P < 0.01. **(A)** Men's accuracy in the Stroop test. **(B)** Men's reaction time in the Stroop test. **(C)** Women's accuracy in the Stroop test. **(D)** Women's reaction time in the Stroop test.

Based on the average 5 min values, different interventions did not significantly affect mental activity in terms of accuracy and reaction time in both men and women, as shown in Table 2 and Figure 3. However, men's accuracy rate in the Stroop test was highest after SLOW among all methods, and it was significantly higher after SLOW than after MUSIC and FAST at the 5th min (SLOW vs. MUSIC, Wald χ2 = 9.44, *P* = 0.011, g = 0.622; SLOW vs. FAST, Wald χ2 = 16.9, *P* < 0.001, g = 0.863). This indicates that the SLOW intervention has a better potential to sustain the ability of inhibition control in men. Additionally, men's reaction time after the SLOW was stable with a slight decrease through 5 min task, and became the shortest at the 5th min, which indicates participants' working speed after SLOW was sustained well relative to after other three methods.

**Table 2:** Results of executive function.

**Interventions**	**1st min**	**2nd min**	**3rd min**	**4th min**	**5th min**	**Averaged 5 min**	**Wald χ^2^**	***P***
**Men's accuracy of the Stroop test (percentage of correct answers, %)**								
Music	94.7 ± 6.0	95.7 ± 3.9	95.4 ± 4.0	95.6 ± 5.5	92.6 ± 7.8 ^*^	94.8 ± 4.4	5.871	0.118
Mindful	95.0 ± 4.2	94.7 ± 3.9	95.0 ± 3.9	96.3 ± 3.9	94.7 ± 3.4	95.2 ± 2.9		
Slow	95.1 ± 3.7	96.0 ± 3.7	95.8 ± 4.7	94.3 ± 4.9	96.4 ± 3.7 ^*#^	95.5 ± 3.1		
Fast	93.8 ± 4.8	93.9 ± 5.2	93.8 ± 7.9	93.4 ± 6.8	92.1 ± 6.0 ^#^	93.4 ± 4.9		
^*^Slow vs. Music (5th min), *P* = 0.011, Hedges' g = 0.622.								
^#^SLOW vs. FAST (5th min), *P* < 0.001, Hedges' g = 0.863.								
**Men's reaction time of the Stroop test (ms)**								
Music	705 ± 139	720 ± 129	725 ± 137	711 ± 119	730 ± 1	718 ± 119	1.889	0.596
Mindful	734 ± 147	718 ± 127	717 ± 123	710 ± 102	743 ± 134	724 ± 116		
Slow	728 ± 134	730 ± 141	720 ± 127	727 ± 156	710 ± 110	723 ± 125		
Fast	725 ± 162	728 ± 166	736 ± 153	742 ± 147	754 ± 149	737 ± 146		
**Women's accuracy of the Stroop test (percentage of correct answers, %)**								
Music	95.7 ± 4.0	96.8 ± 3.4	94.7 ± 5.0	95.0 ± 4.0	93.5 ± 7.4	95.1 ± 4.0	1.008	0.799
Mindful	94.2 ± 5.1	95.9 ± 3.5	95.7 ± 4.3	95.9 ± 4.1	94.8 ± 4.8	95.3 ± 3.3		
Slow	95.1 ± 3.5	95.6 ± 3.4	94.7 ± 3.9	95.5 ± 3.5	94.6 ± 3.9	95.1 ± 2.6		
Fast	95.1 ± 6.1	94.6 ± 8.0	94.6 ± 6.2	94.4 ± 5.6	94.5 ± 5.5	94.6 ± 5.5		
**Women's reaction time of the Stroop test (ms)**								
Music	717 ± 101	702 ± 102	681 ± 75	707 ± 92	708 ± 82	703 ± 78	0.271	0.965
Mindful	694 ± 93	705 ± 96	706 ± 105	719 ± 112	708 ± 76	706 ± 86		
Slow	705 ± 82	705 ± 78	705 ± 104	716 ± 105	702 ± 97	707 ± 82		
Fast	700 ± 99	691 ± 90	705 ± 86	707 ± 107	716 ± 84	704 ± 84		

MUSIC, listening to music; MINDFUL, mindful breathing; SLOW, slow breathing; FAST, fast breathing.

MINDFUL and MUSIC show comparable results in executive function in both women and men. Regarding FAST, clear trends were showing a gradual decrease in accuracy rate and a gradual increase in reaction time in both women and men, with sharper changes in men. This suggests that FAST did not boost executive function but had an adverse impact on it.

In Table 2, we used the mean and standard deviation instead of the median and interquartile ranges because the accuracy was skewed, not due to outliers, but because participants had higher accuracy. Only the mean and SD could clearly and consistently show the analytic results from GEE and the non-parametric Wilcoxon paired-rank test (we conducted the Wilcoxon paired-rank test to confirm the results from GEE).

### 3.3. Respiration rate

One woman's respiration rate was not recorded successfully; therefore, data from 21 men and 21 women were analyzed. All data were normally distributed. Men did not have missing data; women had 1.1%.

**Figure 4:**
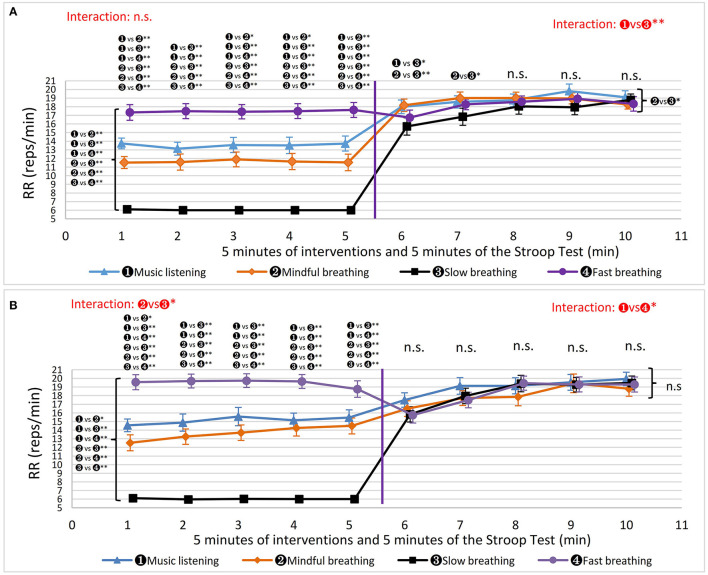
The results of respiration rate (RR). The markers beside the curly brackets, such as “n. s.” or “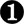 vs 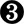
^*^” etc., denote the results of comparison based on the average 5 min values. The markers above each minute present the results of the comparisons based on the values in that minute. The results of interactive effects were colored in red. ^*^P < 0.05, ^**^P < 0.01. **(A)** Men's RR. **(B)** Women's RR.

As depicted in Figure 4A, men's RR was significantly different during different interventions (Wald χ^2^ = 559.3, *P* < 0.001). It was not surprising that the RR of FAST was faster than that of the other methods and SLOW was the slowest. Interestingly, the RR during MINDFUL was significantly lower than that during MUSIC (Wald χ^2^ = 7.168, *P* = 0.007, g = 0.499). During the Stroop test, based on the average 5 min values, RR after SLOW was significantly lower than after MINDFUL (Wald χ^2^ = 6.982, *P* = 0.049, g = 0.382). There was a significant interaction effect on RR during the Stroop test after SLOW and after MUSIC (Wald χ^2^ = 15.9, *P* = 0.019). RR at the 1st min after SLOW was significantly lower than after MUSIC (*P* = 0.010, g = 0.544) and MINDFUL (*P* = 0.001, g = 0.609), and the 2nd min after SLOW was also lower than MINDFUL at the 2nd min (*P* = 0.043, g = 0.542). There were no significant differences at and after the 3rd min. According to Figure 4A and the results obtained, it is evident that 5 min of SLOW reduced RR during stressful conditions for a longer duration than MUSIC and MINDFUL, although not beyond 5 min. From performing intervention to taking the Stroop test, the average 5 min values for RR increased significantly in MUSIC (13.5 ± 3.62 vs. 18.8 ± 3.03, t = −6.68, *P* < 0.001, g = 0.542), in MINDFUL (11.6 ± 3.98 vs. 18.7 ± 2.60, t = −6.80, *P* < 0.001, g = 2.105), and in SLOW (6.02 ± 0.09 vs. 17.5 ± 3.57, t = −14.7, *P* < 0.001, g = 4.516). However, RR after FAST did not change significantly (17.5 ± 4.00 vs. 18.2 ± 2.96, t = −0.70, *P* = 0.490, g = 0.198).

The changes in women's RR are shown in Figure 4B. During the intervention periods, the RR was significantly different between interventions using the average 5 min values (Wald χ^2^ = 841.6, *P* < 0.001). Similar to men's results, RR during MINDFUL was also significantly lower than during MUSIC (Wald χ^2^ = 6.509, *P* = 0.011) in women. Furthermore, there are significant interactions between MINDFUL and SLOW (Wald χ^2^ = 16.8, *P* = 0.013) due to the increase of RR during MINDFUL. During the Stroop test, the results were different from those of men, as RR after various interventions were not different in average 5 min values, nor at each minute, except for the interactive effect after MUSIC and after FAST (Wald χ^2^ = 16.8, *P* = 020). From performing intervention to taking the Stroop test, RR increased significantly in MUSIC (14.9 ± 3.8 vs. 19.0 ± 3.9, z = −3.32, *P* < 0.001, g = 1.065), in MINDFUL (13.6 ± 4.1 vs. 18.1 ± 3.8, z = −3.56, *P* = 0.001, g =1.138), and in SLOW (6.00 ± 0.1 vs. 18.4 ± 3.5, z = −4.02, *P* = 0.001, g = 5.008). Similar to men, RR during FAST did not change significantly (19.6 ± 3.9 vs. 18.3 ± 3.5, z = −1.91, *P* = 0.0559, g = 0.351).

### 3.4. Oxygen saturation

SpO_2_ was not recorded successfully for one man and one woman. Therefore, 20 men and 21 women were included in this analysis. The data from both men and women were normally distributed. Men had 5.8% missing values, whereas women had 3.7%.

**Figure 5:**
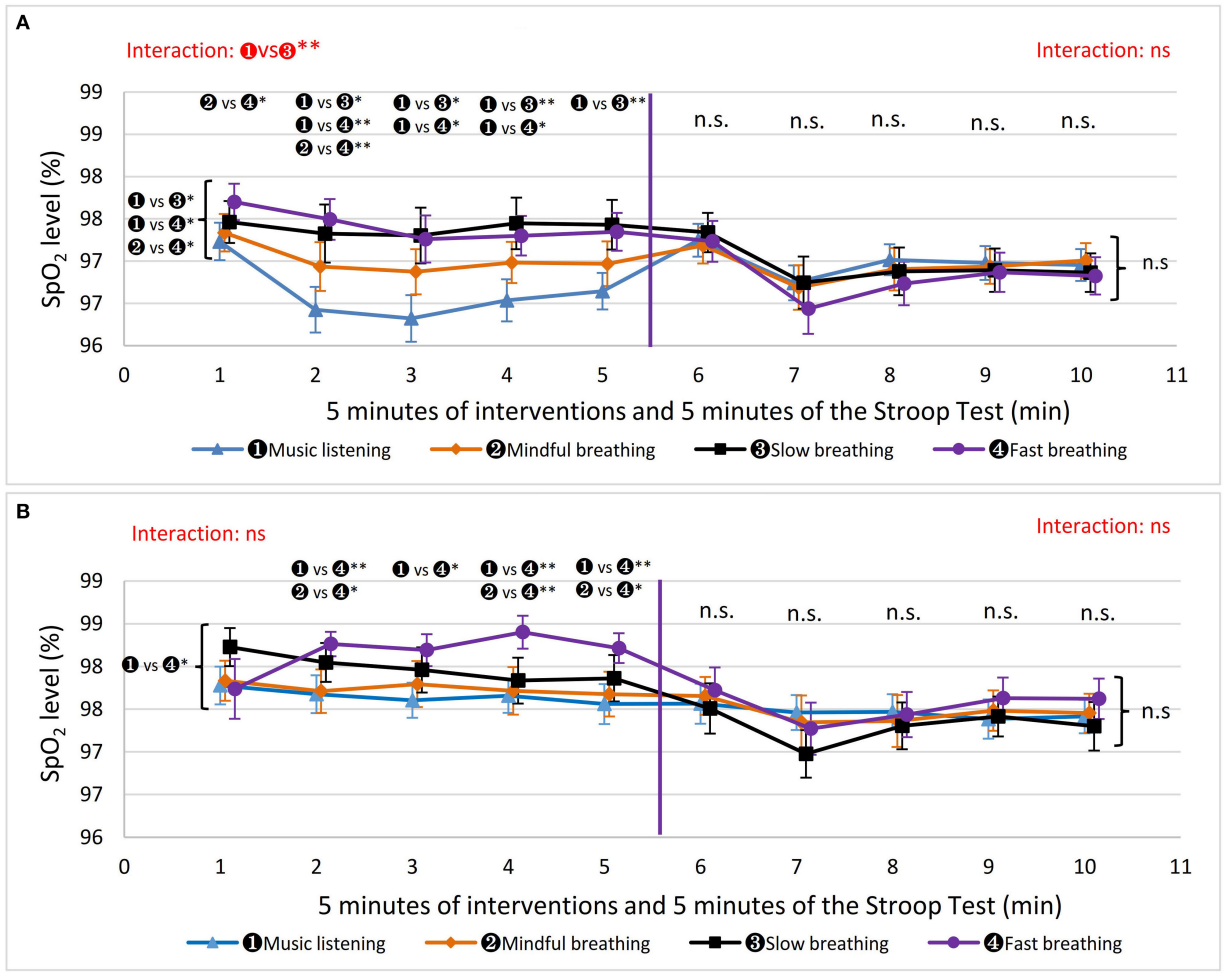
The results of oxygen saturation (SpO2). The markers beside the curly brackets, such as “n. s.” or “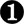 vs 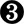
^*^” etc., denote the results of comparison based on the average 5 min values. The markers above each minute present the results of the comparisons based on the values in that minute. The results of interactive effects were colored in red. ^*^P < 0.05, ^**^P < 0.01. **(A)** Men's SpO2. **(B)** Women's SpO2.

For men, during the interventions, the SpO_2_ was significantly different between MUSIC and SLOW (96.6 ± 0.9 vs. 97.4 ± 1.3, Wald χ^2^ = 9.53, *P* = 0.012, g = 0.716), MUSIC and FAST (96.6 ± 0.9 vs. 97.4 ± 1.0, Wald χ^2^ = 8.79, *P* = 0.015, g = 0.841), and MINDFUL and FAST (97.0 ± 1.1 vs. 97.4 ± 1.0, Wald χ^2^ = 7.64, *P* = 0.023, g = 0.381) (Figure 5A). In addition, there was an interactive effect between MUSIC and SLOW (Wald χ^2^ = 21.2, *P* = 0.002). In contrast, there was no significant difference between MUSIC and MINDFUL (96.6 ± 0.9 vs. 97.0 ± 1.1, Wald χ^2^ = 0.31, *P* = 0.502, g = 0.398). During the Stroop test, different interventions did not influence SpO_2_ significantly for the average 5 min values and at each minute. SpO_2_ decreased significantly from performing FAST to taking the Stroop test (97.4 ± 1.03 vs. 96.7 ± 0.99, t = 2.72, *P* = 0.014, g = 0.693). The effect of the interventions lasted for 1 min during the Stroop test, as SpO_2_ dropped significantly from the 2nd minute after MUSIC (*P* < 0.001, MINDFUL (*P* < 0.001), SLOW (*P* = 0.016), and FAST (*P* < 0.001).

For women, during the different interventions, only MUSIC and FAST influenced SpO_2_ differently (97.7 ± 0.95 vs. 98.2 ± 0.73, Wald χ^2^ = 9.46, *P* = 0.012, g = 0.590) (Figure 5B). During the Stroop test, different interventions did not influence SpO_2_ significantly. From performing intervention to taking the Stroop test, SpO_2_ decreased significantly after SLOW (97.9 ± 1.07 vs. 97.3 ± 1.17, t = 3.17, *P* = 0.005, g = 0.535) and FAST (98.2 ± 0.73 vs. 97.5 ± 1.15, t = 3.00, *P* = 0.007, g = 0.727). During the Stroop test, SpO_2_ decreased significantly from the 1st min to the 2nd min only after SLOW (*P* = 0.008).

### 3.5. End-tidal carbon dioxide partial pressure

Data were not recorded successfully for one man and two women. Therefore, data from 20 men and 20 women were analyzed. All data had a normal distribution except during the Stroop test after FAST in women. There were 6% missing data from men and 2.5% from women.

**Figure 6:**
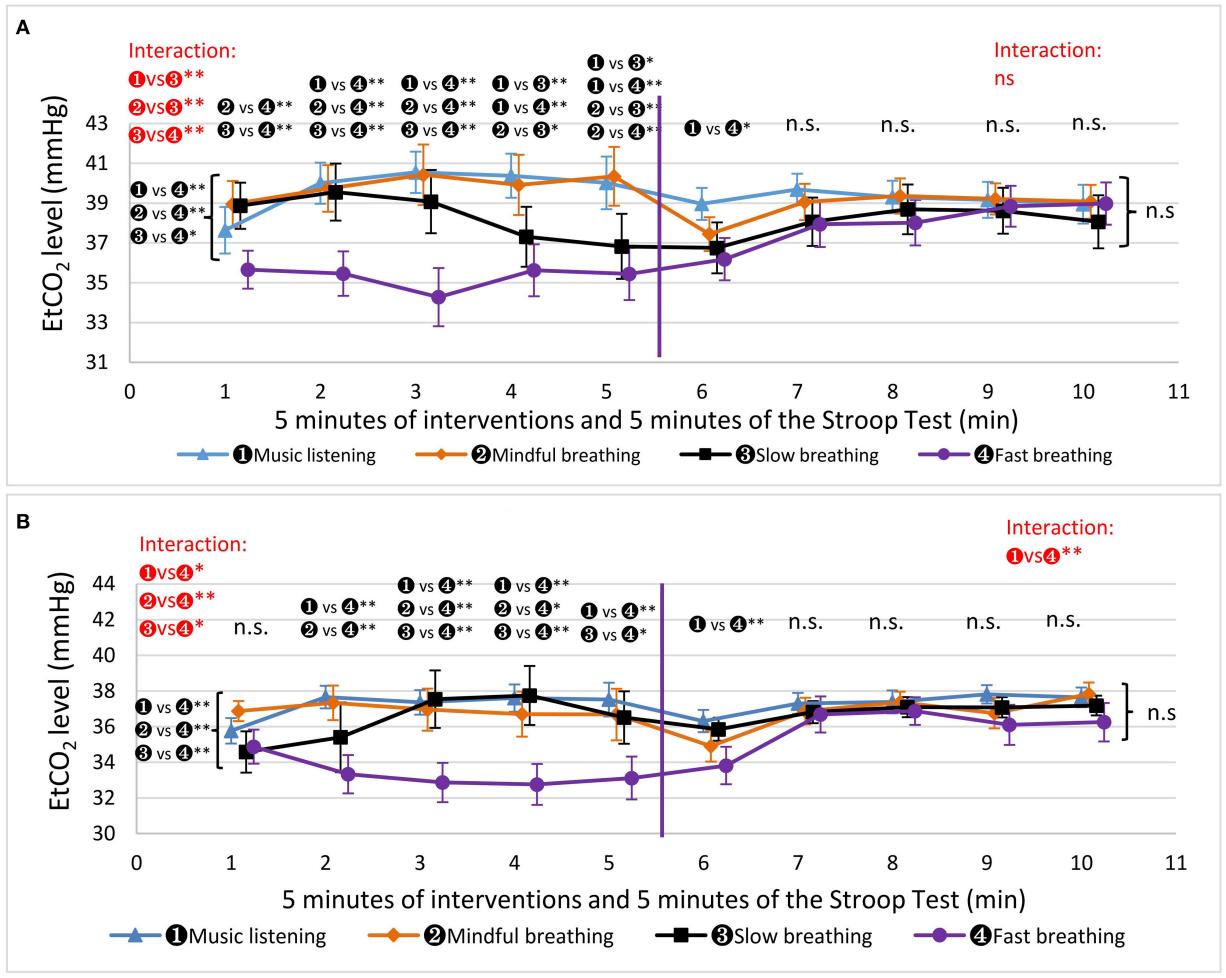
The results of end-tidal carbon dioxide partial pressure (EtCO2). The markers beside the curly brackets, such as “n. s.” or “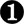 vs 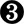
^*^” etc., denote the results of comparison based on the average 5 min values. The markers above each minute present the results of the comparisons based on the values in that minute. The results of interactive effects were colored in red. ^*^P < 0.05, ^**^P < 0.01. **(A)** Men's EtCo2. **(B)** Women's EtCo2.

The results for men are shown in Figure 6A. During the different interventions, EtCO_2_ was significantly different, using the average 5 min values, between FAST and MUSIC (35.3 ± 5.29 vs. 39.9 ± 4.82, Wald χ^2^ =18.19, *P* < 0.001, g = 0.929), FAST and MINDFUL (35.3 ± 5.29 vs. 40.0 ± 5.80, Wald χ^2^ =19.22, *P* < 0.001, g = 0.865), and FAST and SLOW (35.3 ± 5.29 vs. 38.5 ± 6.36, Wald χ^2^ = 7.02, *P* = 0.032, g = 0.547). In addition, there were significant interactive effects between SLOW and MUSIC (Wald χ^2^ = 48.8, *P* < 0.001), between SLOW and MINDFUL (Wald χ^2^ = 26.4, *P* < 0.001), and between SLOW and FAST (Wald χ^2^ = 26.2, *P* < 0.001). Notably, EtCO_2_ after SLOW started to reduce from the 2nd min; it was significantly lower than that of MUSIC and MINDFUL at the 4th min (37.0 ± 6.70 vs. 40.6 ± 5.12, *P* = 0.004, g = 0.547; 37.0 ± 6.70 vs. 39.8 ± 7.01, *P* = 0.036, g = 0.408), and at the 5th min (37.0 ± 6.70 vs. 40.2 ± 6.07, *P* = 0.005, g = 0.472; 37.0 ± 6.70 vs. 40.2 ± 6.88, *P* = 0.003, g = 0.447). During the Stroop test, different interventions did not influence EtCO_2_ significantly based on the average 5 min values. Only at the 1st min, EtCO_2_ after FAST was significantly lower than that after MUSIC (39.0 ± 3.76 vs. 36.2 ± 4.88, *P* = 0.046, g = 0.642). From performing intervention to taking the Stroop test, EtCO_2_ increased significantly only in FAST (35.3 ± 5.29 vs. 37.9 ± 4.68, t = −3.30, *P* = 0.023, g = 0.521).

The results for women are shown in Figure 6B. EtCO_2_ during FAST (33.2 ± 4.18) was significantly lower than that during the other three interventions (MUSIC: 37.1 ± 2.87, Wald χ^2^ = 18.66, *P* < 0.001, g = 1.088; MINDFUL: 36.7 ± 4.43, Wald χ^2^ = 10.74, *P* = 0.004, g = 0.813; SLOW: 35.9 ± 5.70, Wald χ^2^ = 12.32, *P* = 0.002, g = 0.540), and there were no significant differences between MUSIC, MINDFUL and SLOW. Furthermore, there were significant interactive effects between FAST and MUSIC (Wald χ^2^ = 17.0, *P* = 0.011), between FAST and MINDFUL (Wald χ^2^ = 25.2, *P* < 0.001), and between FAST and SLOW (Wald χ^2^ = 16.4, *P* = 0.013). During the Stroop test, different interventions did not influence EtCO_2_ significantly for the average 5 min values. However, there was an interactive effect after MUSIC and after FAST (Wald χ^2^ = 30.0, *P* < 0.001), and EtCO_2_ after FAST was significantly lower than that after MUSIC (33.8 ± 5.25 vs. 36.3 ± 2.95, *P* = 0.002, g = 0.587) only in the 1st min. From performing the intervention to the Stroop test, comparing the average 5 min values, EtCO_2_ increased significantly only for FAST (33.2 ± 4.18 vs. 35.8 ± 5.00, t = −3.49, *P* = 0.003, g = 0.564).

### 3.6. Preferred intervention

**Figure 7:**
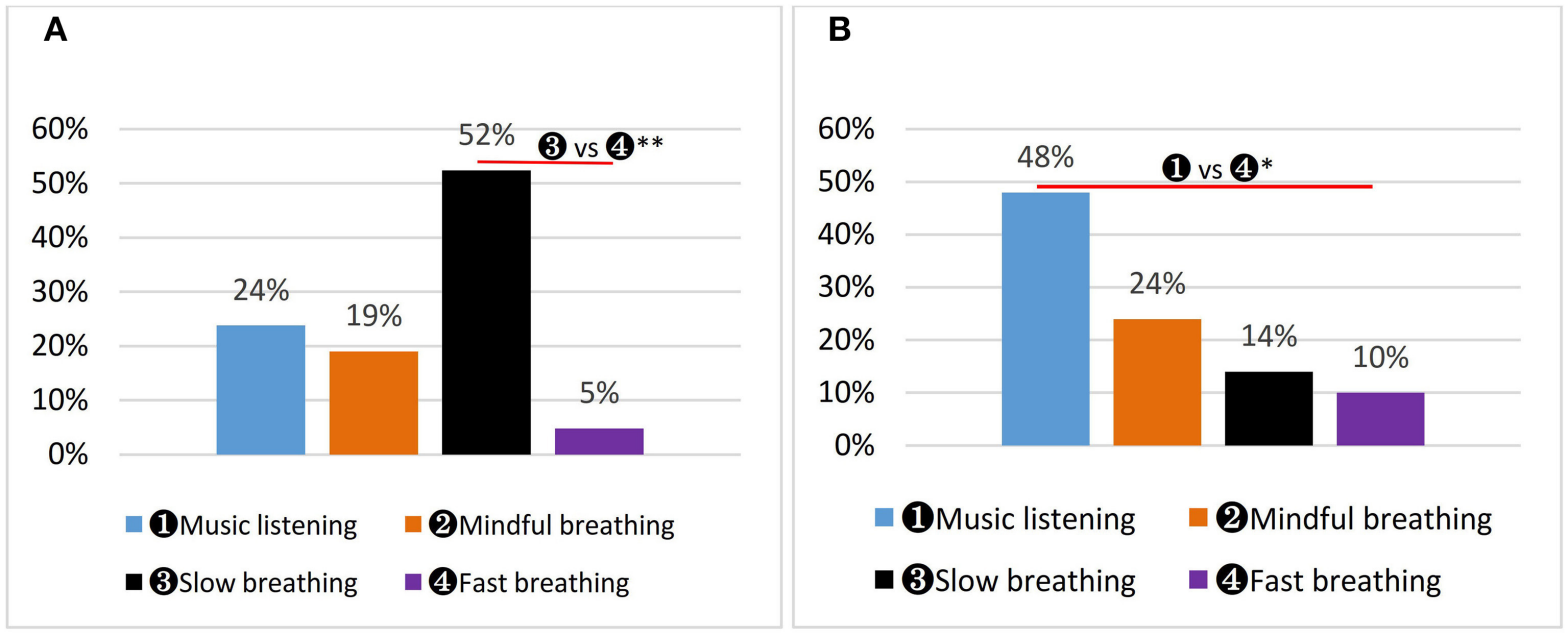
Participants' preferred intervention. ^*^P < 0.05, ^**^P < 0.01. **(A)** Men's preferred intervention. **(B)** Women's preferred intervention.

As shown in Figure 7A, 52% (*n* = 11) of the male participants preferred SLOW, 24% (*n* = 5) MUSIC, 19% (*n* = 4) MINDFUL, and 5% (*n* = 1) FAST. Statistically, the percentage of participants who preferred SLOW was significantly higher than that of participants who preferred FAST (χ^2^ = 11.37, *P* = 0.004). For the female participants, 48% (*n* = 10) preferred MUSIC, 24% (*n* = 5) MINDFUL, 14% (*n* = 3) SLOW, and 10% (*n* = 2) FAST (Figure 7B). One woman could not firmly choose a favorite, so she did not respond. The percentage of female participants who preferred MUSIC was significantly higher than those who preferred FAST (χ^2^ = 7.19, *P* = 0.004).

## 4. Discussion

The purpose of the current study was to test which method (within breathing techniques and music listening) is better for body relaxation and work efficacy such that it could be used alone or in combination with physical activity during work breaks. To the best of our knowledge, this is unprecedented research to investigate the effect of breathing techniques on muscle tension, even though we found different breathing methods did not influence muscle tension significantly. However, MINDFUL tended to be better at muscle tension reduction under stressful conditions for men, and SLOW showed superior potential over other methods on executive function. In addition, the results of RR, SpO_2_, and EtCO_2_ provided many interesting and meaningful physiological findings.

### 4.1. Muscle tension

One explanation for increased muscle tension under stress is that the elevated sympathetic activity initiates the release of catecholamines, which further increases muscle tension (Melin and Lundberg, 1997). Another study suggested that stress-induced hyperventilation causes excessive exhalation of CO_2_, which increases pH and facilitates muscle contraction (Schleifer et al., 2002). Mental stress in computer work increased muscle tension and brief MINDFUL or SLOW was able to reduce stress (Lundberg et al., 1999; Karthikeyan et al., 2012; Beng et al., 2016). Therefore, we investigated the influence of different breathing methods and MUSIC on muscle tension.

Unexpectedly, but meaningfully, we found that 5 min of MINDFUL, SLOW, FAST, and MUSIC did not influence upper trapezius activity when taking the stressful psychological task, the Stroop test. To verify the results, successful stress induction was confirmed, as RR, a stress indicator, increased when performing the Stroop test. Second, two sEMG normalization methods (normalized from the mean and baseline) were performed, and there were no significant differences between each intervention.

According to the mean values, men's muscle tension was the lowest after MINDFUL from both the right and left sides of the upper trapezius, and the muscle tension was higher after SLOW than after MINDFUL and MUSIC. This implies that it might be difficult to relax physically while being energized mentally. The muscle tension in women was lower after MUSIC and FAST than that after SLOW and MINDFUL, while the lowest was after FAST. Oddly, FAST was the least favorable method. In addition, the muscle tension was inconsistent between the left and right sides. For example, men's muscle tension was the highest after FAST from the right side and the highest after SLOW from the left side. These odd and inconsistent results might be due to the influence of posture. We noticed that some participants' muscle tension changed substantially when their posture was altered. We strictly specified where the participants' arms were to rest. However, there were movements of the upper body during the Stroop test. Cohen et al. (1983) also stressed “EMG is influenced by many other factors, apart from stress. Static factors like body morphology and dynamic factors like posture influence the recorded EMG signals possibly even more than stress.”

### 4.2. Executive function

Slow breathing has been shown to have better accuracy, but not reaction time, on the Stroop test compared to that of watching a neutral TV program (Laborde et al., 2022). In the present study, SLOW showed greater potential for sustaining executive function in men, as the accuracy rate during the average 5 min values was highest after slow breathing, with the accuracy rate in the 5th min being significantly higher after slow breathing compared to music listening and fast breathing, and the reaction time being shortest after slow breathing. A resonance model proposed 6 reps/min for SLOW as being the most beneficial. In this model, SLOW was related to heart rate, blood pressure, baroreflex modulation, and resonance characteristics of the cardiovascular system (Lehrer and Gevirtz, 2014). Laborde et al. (2019) summarized that these processes could strengthen homeostasis, improve gas exchange, and increase vagal afferents. Our findings support this model as SpO_2_ during SLOW was higher than during MUSIC.

Respiration interacts with the autonomic nervous system, as inhalation is associated with sympathetic activity and exhalation with parasympathetic activity (Jerath et al., 2006). SLOW increases parasympathetic outflow (Kromenacker et al., 2018), while decreased sympathetic activity and increased parasympathetic activity is likely associated with better cognitive performance (Forte et al., 2019). In the present study, SLOW was set as 4 s of inhalation and 6-s exhalations, which was meant to increase parasympathetic activity, and the improved cognitive function might be caused by the activation of the parasympathetic outflow. As mentioned in the introduction, Laborde et al. (2022) observed an increased RMSSD in HRV (represents cardiac vagal activity/pare-sympathetic activity) during slow breathing (4.5-s inhalation and 5.5-s exhalation) and improved executive function after slow breathing. However, they found that improved executive function (compared to watching TV) was not mediated by RMSSD using bootstrapped mediation analyses (Laborde et al., 2022). Nevertheless, while slow breathing reduces stress (Zaccaro et al., 2018), the Stroop test was stressful, and even mild acute uncontrollable stress can cause a rapid and dramatic loss of prefrontal cognitive abilities (Arnsten, xbib2009). SLOW may improve executive function by alleviating the effects of stress and maintaining prefrontal neuronal function during stressful psychological tasks. The human body is a complex system. Oxygenation and respiration rate might also be the medicative factors, which will be discussed in the next part, “cardiorespiratory Activity.”

Regarding the results in women, the measured interventions did not significantly affect their executive function. Women have shorter diaphragms, higher positions of the sternum, a greater inclination of the ribs, and more chest movement than men during regular breathing (García-Martínez et al., 2016; LoMauro and Aliverti, 2018). Additionally, cultural influences may lead women to contract their abdominal muscles in order to appear more slender. Therefore, we suggest that women might need a longer time than men to practice SLOW (with obvious abdominal movement) to gain the same effect as men.

The effect of 5 min of MINDFUL on the performance of the executive test was not significantly different from MUSIC, even though the RR during MINDFUL was significantly lower than during MUSIC, and the SpO_2_ was relatively higher than that of MUSIC.

Fast breathing was the least effective intervention according to the Stroop test results. We did not find a boost in mental ability, as we hypothesized, due to increased pH and neuronal excitability. The acute effects we found were not consistent with chronic effects, as a previous study showed that 12 weeks of fast yoga breathing significantly improved healthy young adults' executive function (Sharma et al., 2014). Fast breathing often accompanies high-anxiety states, and it is possible that voluntarily increasing the breathing rate can trigger mechanisms similar to those triggered by anxiety (Masaoka and Homma, 1997; Homma and Masaoka, 2008). In our study, some men felt nervous after FAST, which might be due to mild hyperventilation and that decreased performance in the Stroop test.

Additionally, as inhalation and exhalation trigger different autonomic nervous activities, we suggest conducting a study to compare the different effects of inhalation/exhalation ratios on executive function.

### 4.3. Cardiorespiratory activity

The respiration rate, SpO_2_, and EtCO_2_ were evaluated to monitor physiological responses and explain the results. Although not the primary focus, there were new findings, and some could meaningfully strengthen the results of previous studies.

Both men's and women's RR during MINDFUL were lower than during MUSIC, which is in line with the finding that MINDFUL tended to result in a lower rate of respiration (Hunt et al., 2021). Paying attention to breathing strengthens breathing activity, even though we do not intend to influence it. In the Stroop test, men's RR after SLOW was the lowest when compared to that of the other interventions. We propose two physiological mechanisms to explain these results. First, SLOW increased ventilation volume, as shown in Figure 6A, which activated the regulation center in the medulla oblongata and inhibited the respiration rate (Kandel et al., 2013). Second, SLOW activated the parasympathetic nervous system (increased vagal tone) (Radaelli et al., 2004). The effect after SLOW decreased stress during the Stroop test, so the respiration rate did not increase as much as it did after MINDFUL and MUSIC. The second proposed mechanism was strengthened by the results of FAST. FAST increased ventilation more than SLOW, and theoretically, it should inhibit RR more than SLOW during the Stroop test, but it did not. Therefore, the reduction in RR after SLOW during the Stroop test was more likely due to the increased parasympathetic activity. Additionally, since RR is an indicator of stress, our findings are consistent with a previous report (Hunt et al., 2021) where SLOW was more effective than MINDFUL in stress reduction.

Oxygen saturation was higher during SLOW than during MUSIC and higher during FAST than during MUSIC and MINDFUL. There were slight differences in the mean and standard deviation between the SLOW and FAST groups. However, EtCO_2_, directly influenced by ventilation volume, was significantly different between the SLOW and FAST groups. These results confirmed SLOW's superior respiration efficiency compared to FAST, as hemoglobin oxygenation during SLOW can reach levels as high as those during FAST, but with less ventilation.

Consistently, the SpO_2_ alterations by different interventions did not last longer than 1 min during the Stroop test. A similar result was also found in EtCO_2_, where only FAST maintained EtCO_2_ higher than MUSIC in the 1st min. These two findings indicate that the carryover effect of breathing on SpO2 and EtCO_2_ was short.

### 4.4. Breathing method preference

Subjective preference should be a serious consideration when choosing a breathing technique because it directly reflects participants' physiological state. In this study, neither men nor women liked FAST, most men liked SLOW, and most women liked MUSIC. Participants chose according to their general subjective feelings of relaxation and working efficiency. The results mean most women felt better after MUSIC, whereas most men felt better after SLOW. Although 52% of men preferred SLOW, there were still 24% who preferred MINDFUL. Similarly, although 48% of women preferred MUSIC, there were still 24% who preferred MINDFUL. These findings demonstrate the diversity of participants' physiological responses after using different methods. Our findings can be used as a reference, but people should choose a breathing method (or music) according to their mental and physical states.

### 4.5. Limitations

This study has at least four limitations. First, we noticed that some participants' muscle tension changed substantially when their posture was altered. This phenomenon was difficult to control because some participants unconsciously adjusted their posture during the Stroop test. Second, 5 min of MINDFUL might be too short to generate enough of an effect to influence cognitive function and muscle tension, although a previous study found that it significantly reduced distress scores. Third, we did not ask participants the specific reasons for their preference ratings. Fourth, although we used the Latin Square Williams design to balance the influence of the carryover effect, there should be still a carryover effect between each intervention. Future studies might consider testing interventions on different days.

## 5. Conclusion

Different breathing methods and MUSIC did not significantly influence muscle tension. Men's executive function tended to be better after SLOW than after MUSIC and FAST, whereas women's executive function did not depend on the intervention. SLOW's superior respiration efficiency on SpO_2_ and inhibition of RR might be the causes of the men's results. Most women preferred MUSIC, and most men preferred SLOW, while FAST was least favorable.

## Data availability statement

The data that support the findings of this study are available upon request from the corresponding author JX.

## Author contributions

OR, JX, and W-ML contributed to the conceptualization, methodology, and formal analysis. W-ML, JX, F-FR, Z-SC, and Z-MB contributed to data collection and/or processing. W-ML wrote the original draft. OR, JX, F-FR, Z-SC, C-RL, and Z-MB reviewed and edited the manuscript. OR and JX supervised the study, provided equipment, and acquired funds. All authors have read and agreed to the published version of the manuscript.
